# Effect of Xanthan Gum on the Rheological Behavior and Microstructure of Sodium Caseinate Acid Gels

**DOI:** 10.3390/gels2030023

**Published:** 2016-09-10

**Authors:** María E. Hidalgo, Mirta Armendariz, Jorge R. Wagner, Patricia H. Risso

**Affiliations:** 1 Departamento de Química-Física, Facultad de Ciencias Bioquímicas y Farmacéuticas, Universidad Nacional de Rosario, Suipacha 531, S2002LRK Rosario, Santa Fe, Argentina; maruhidalgo80@yahoo.com.ar (M.E.H.); mirtaarmendariz@gmail.com (M.A.); 2 Instituto de Física Rosario (IFIR-CONICET-UNR), 27 de Febrero 210 Bis, S2000EKF Rosario, Santa Fe, Argentina; 3 Departamento de Ciencia y Tecnología, Universidad Nacional de Quilmes, CONICET, Roque Sáenz Peña 352, B1876BXD Bernal, Buenos Aires, Argentina; jorge.wagner@unq.edu.ar; 4 Facultad de Ciencias Veterinarias, Universidad Nacional de Rosario, Ovidio Lagos y Ruta 33, 2170 Casilda, Santa Fe, Argentina

**Keywords:** protein-polysaccharide interactions, thermodynamic compatibility, experimental design, digital images analysis

## Abstract

The aim of this work was to study the effect of xanthan gum (XG) on the gelation process of bovine sodium caseinate (NaCAS) induced by acidification with glucono-δ-lactone (GDL) and on the mixed acid gel microstructure. Before GDL addition, segregative phase separation was observed in all the NaCAS-XG mixtures evaluated. The gelation process was analyzed by using a fractional factorial experimental design. The images of the microstructure of the mixed acid gels were obtained by conventional optical microscopy and the mean diameter of the interstices was determined. Both the elastic character and the microstructure of the gels depended on the concentrations of XG added. As XG concentration increased, the kinetics of the gelation process was modified and the degree of compactness and elasticity component of the gel network increased. The microstructure of gels depends on the balance among thermodynamic incompatibility, protein gelation and NaCAS-XG interactions.

## 1. Introduction

Mixed gels of proteins and polysaccharides have proved to control the texture and stability of food products. Therefore, it is essential to understand the interactions between both biopolymers and how they contribute to the improvement of the physical properties of food [1,2]. The mixture of proteins and polysaccharides in aqueous solutions can result in phase separation, which can be either segregative due to limited thermodynamic compatibility, or associative due to the formation of a complex through weak and nonspecific interactions [2]. Thermodynamic incompatibility, which arises from the low entropy of mixing two polymers, usually occurs near the protein isoelectric point, promoting protein self-association and/or when each polymer has a different affinity for the solvent [3].

Bovine sodium caseinate (NaCAS) is used as an ingredient in a wide range of food products due to its nutritional and functional properties. The acidification of NaCAS generates gel formation near the isoelectric point (pI). The physical properties of NaCAS gels are intimately associated with their chemical properties [4] and with different factors such as pH, temperature and ionic strength [5,6,7]. The use of glucono-δ-lactone (GDL) as a starter of the acidification processes produces a slow reduction in pH and avoids some of the difficulties associated with the use of starter bacteria [8].

Xanthan gum (XG), an anionic polysaccharide produced by *Xanthomonas campestris*, is commonly used in the food industry because of its high water solubility, stability of its aqueous solutions in a wide pH range and its high viscosity [9].

XG primary structure consists of a linear (1-4)-β-d glucose backbone with a charged trisaccharide side chain on each second glucose residue [10]. Although XG is not considered as a gelling agent, some authors have obtained gels from heated XG aqueous solutions [11,12].

Interactions between milk proteins and anionic polysaccharides, in both aqueous solutions and emulsion systems have been reported [13,14,15,16,17,18]. The behavior of NaCAS-XG mixtures in emulsified [19,20,21] and gelled [22] dairy products, and in aqueous solutions [9,21,23] has also been described. On the other hand, whereas mixtures of milk and XG at neutral pH studies have frequently been reported, there is a limited number of studies in the literature concerning the use of XG during acid-induced gelation [22,24,25].

Due to the fact that the domain of protein-polysaccharide interactions is an essential factor in the development of new dairy products, physicochemical and functional properties, such as gelation, are worth exploring in detail. The rheological properties of gels are closely related to gel microstructure and strength. Moreover, rheological properties are strongly affected by gel chemical composition and microstructure, which depend on the type of interactions involved. Therefore, the study of gels is important not only at macro level (rheological behavior), but also at micro scale (protein conformational changes and gel microstructure). The aim of this work was to evaluate the gelation process of NaCAS-XG blends as a model system of acid dairy products, linking the rheological behavior with the microstructure properties of these gels.

## 2. Results and Discussion

### 2.1. Thermodynamic Compatibility of NaCAS–XG Blends

**Figure 1:**
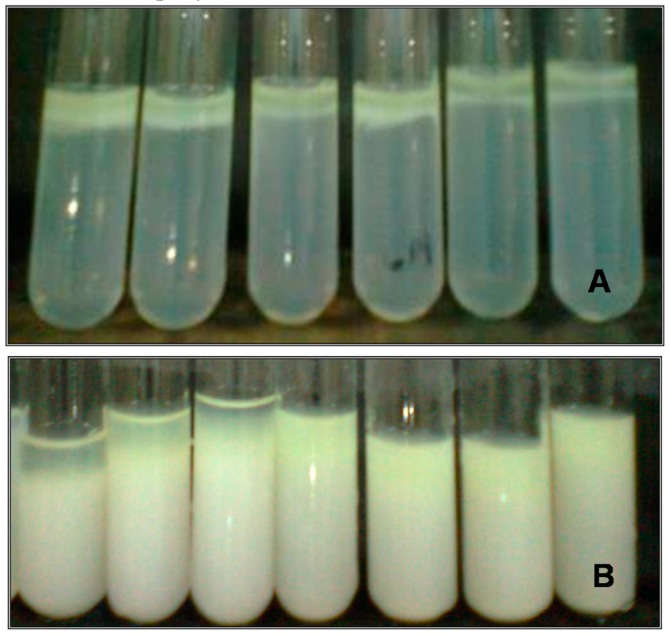
Separation of phases of NaCAS-XG systems due to thermodynamic incompatibility after 24 h (**A**) and 48 h (**B**) of incubation at 35 °C and controlled humidity.

A complete separation of phases was observed in all the cases studied. Therefore, phase diagrams could not be obtained due to the thermodynamic incompatibility expressed throughout the concentration range tested (*C*_XG_ 0–0.45 wt %; *C*_NaCAS_ 0–4 wt %). Figure 1 shows some selected systems. These findings suggest that there is a segregative phase separation, where the bottom phase is rich in protein and the top phase is rich in polysaccharide.

Hemar et al. (2001) have studied NaCAS-XG systems at pH 6.70 and reported a phase micro-separation. These authors have hypothesized that the presence of protein promotes the self-association of XG molecules. At neutral pH, where NaCAS and XG have negative net charges, thermodynamic incompatibility may occur due to repulsive interactions between both biopolymers [21]. Rodd et al. (2000) have also reported the association of xanthan molecules [26].

### 2.2. Protein Conformational Changes

**Figure 2:**
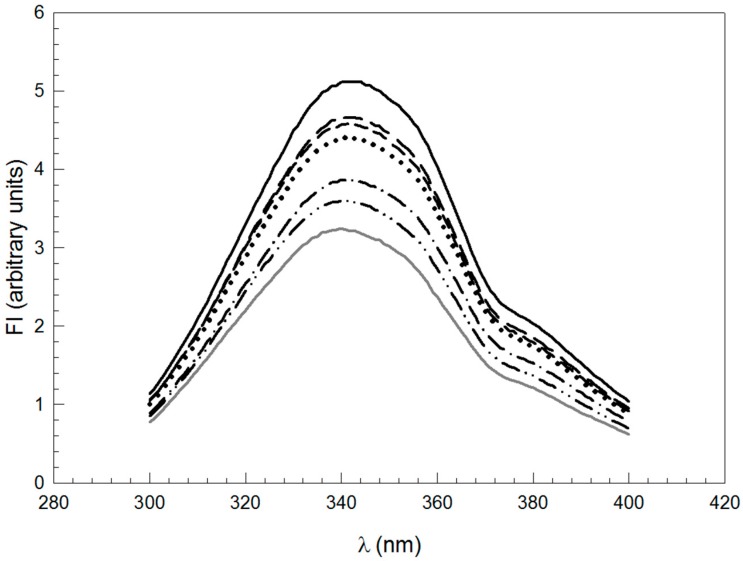
Emission spectra of intrinsic fluorescence (FI) of NaCAS and NaCAS:XG mixtures at different ratios: (**─**) without XG; (‒ ‒) 8:1; (- - -) 6:1; (•••) 4:1; (‒ • ‒) 2:1; (‒ •• ‒) 1:1 and (─) 1:1.5. NaCAS 0.1 wt %, T 35 °C, emission wavelength: λ_em_ = 300–400 nm; excitation wavelength: λ_exc_ = 286 nm.

Figure 2 shows the emission spectra of intrinsic fluorescence of NaCAS and of NaCAS:XG mixtures. An increment in XG concentration produced a decline in intrinsic fluorescence (FI) without changes in emission peaks. This would indicate that, in the presence of the XG, there might be an increased exposure of protein fluorophores to a more polar environment [27].

Qi et al. (2001) reported that the concept of tensegrity can be used to explain the structural interaction of caseins. In the three-dimensional models of caseins, extensions between the curves centered on proline residues provide rigid areas, while the turns and helices represent the most flexible elements [28]. In the presence of XG, a restriction volume for NaCAS particles occurs due to phase micro-separation and this effect might induce NaCAS particle association. Secondary structure zones, which are incapable of tertiary folds, can lead to self-association, but due to the compromise between tension and flexibility, no hydrophobic compression occurs and the rest of the protein configuration opens and hydrates, which might lead to an exposition of protein fluorophores to the polar medium.

### 2.3. Effect of XG on the Viscosity of the Medium

**Figure 3:**
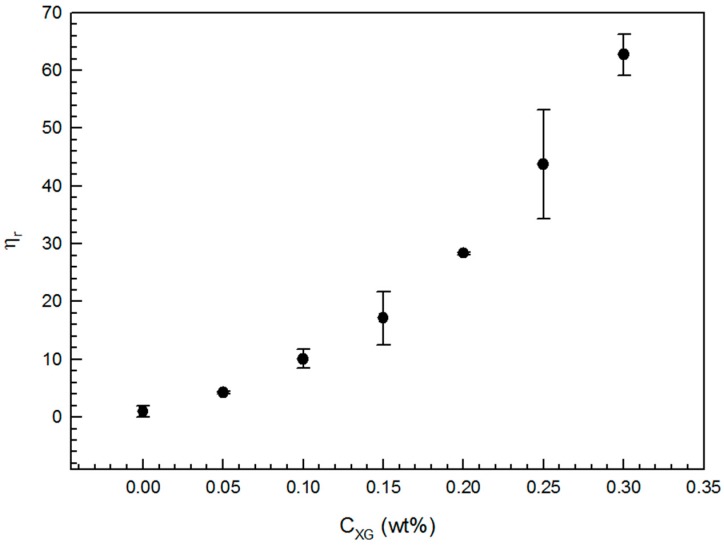
Variation of η_r_ as a function of XG concentration (*C*_XG_: 0–0.30 wt %); Cone spindle CPE-42, Shear rate: 11.54 s^−1^; T 35 °C.

XG has been extensively studied and widely used in food products due to its high viscosity and to its pseudo-plastic behavior in aqueous solutions. In addition, the protein aggregation process is limited by particle diffusion. Thus, the effect of XG addition on viscosity in the absence of protein was determined. Figure 3 shows that η_r_ exponentially increased with the increment of polysaccharide concentration in the range of concentrations studied. η_r_ was high enough even at low concentrations of XG and, at high XG concentration, the viscosity of the system was considerably high, which is consistent with the results obtained by Hemar et al. [21].

### 2.4. Rheological Properties of NaCAS Acid Gels in the Presence of XG

In the absence and presence of XG, the rheological behavior of NaCAS aqueous solutions (3 and 5 wt %) was studied. In order to evaluate the significance of the variables assayed (T, R, C_XG_ and C_NaCAS_), a fractional factorial design 2^4-1^ was performed. The response variables were t_gel_, pH_gel_ and G’_max_. Table 1 shows the coded and uncoded variable values and the responses obtained and Table 2 shows the coefficients and *p*-values obtained.

By response surface adjustment, Equations (1)–(3) were obtained, which contain the model for the variation of t_gel_, pH_gel_ and G’_max_, respectively (the quadratic terms are not taken into account because of *p* >> 0.05).

(1) $$t_{\mathrm{gel}}\;=\;15.60\;-\;12.86\;C_{\mathrm{XG}}\;-\;7.54\;R\;-7.23\;T\;+\;8.16\;C_{\mathrm{XG}}\;R\;+\;8.72\;C_{\mathrm{XG}}\;T\;+\;5.78\;R\;T$$

**Figure 4:**
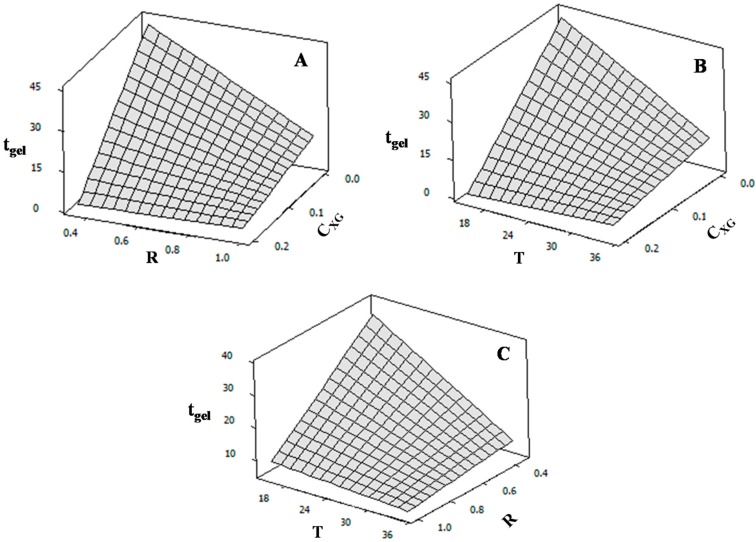
Response surface plots: (**A**) t_gel_ (min) as a function of GDL mass fraction/NaCAS mass fraction ratio (R) and xathan gum concentration (C_XG_: wt %); (**B**) t_gel_ (min) as a function of temperature (T: °C) and xathan gum concentration (C_XG_: wt %) and (**C**) t_gel_ (min) as a function of GDL mass fraction/NaCAS mass fraction ratio (R) and T (°C).

The increase in *C*_XG_, *R* and *T* produced a decrease in *t*_gel_. Since medium viscosity substantially increased as *C*_XG_ increased (Figure 3), the NaCAS particle diffusion was restricted or limited; thus, the formation of NaCAS-XG acid gel network was retarded. If the amount of GDL added increases (*R* rises), the time at which NaCAS particles become unstable and begin to aggregate decreases because the rate at which the pH becomes lower increases. Braga et al. (2006) also reported this fact for caseinate acid gels [29]. *T* causes two effects on the *t*_gel_. On one hand, an increase in *T* favors the hydrophobic interactions involved in the gelation process. On the other hand, *T* increases GDL hydrolysis rate and, hence, the rate at which pH becomes lower. Considering these results, the kinetic of NaCAS acid gelation could be modified through changes on *T* and *R*. Figure 4 shows the response surface plots obtained.

(2) $${\mathrm{pH}}_{\mathrm{gel}}\;=\;5.63\;+0.51\;C_{\mathrm{XG}}\;-\;0.09\;R$$

**Figure 5:**
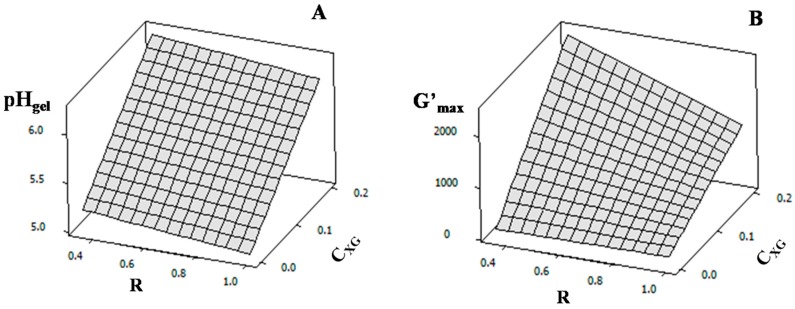
Response surface plots: (**A**) pH_gel_ as a function of GDL mass fraction/NaCAS mass fraction ratio (R) and xathan gum concentration (C_XG_: wt %) and (**B**) G’_max_ (Pa) as a function of R and xathan gum concentration (C_XG_: wt %).

An increase in *C*_XG_ causes an increase in pH_gel_ while an increment in *R* diminishes it (Figure 5A). As mentioned above, NaCAS particles lose their colloidal stability because of the induction of phase micro-separation of XG and NaCAS. This phase micro-separation increases as *C*_XG_ increases, making the aggregation process begin earlier. Then, NaCAS particles form gels at a higher pH. On the other hand, in order to start the gelation process, the electrostatic repulsion due to negative surface electric potential of NaCAS particles must be removed. This is achieved by binding protons to protonable NaCAS residues, which results from gluconic acid dissociation. Therefore, as *R* increases, a higher concentration of protons is able to electrostatically destabilize the protein (lower pH). Braga et al. (2006) reported that pH_gel_ was about 5.0–5.1 for caseinate acid gels in a range of *R* of 0.18–0.36, which is lower than the one used in this work [30].

(3) $$G{’}_{\max}=\;1002.2\;+\;832.1\;C_{\mathrm{XG}}\;-\;290.3\;R\;-\;301.3\;C_{\mathrm{XG}}\;R$$

Before GDL addition, at pH~6.8, NaCAS particles have a high negative net charge and repulsive interactions between NaCAS and XG predominate over attractive interactions. Then, the preferential concentration of NaCAS in one phase and of XG in another phase takes place. After GDL addition, during the acidification process, the protein negative net charge decreases while XG net charge does not change. As a consequence, the attractive interactions between the polyanionic XG and the less negatively charged NaCAS particles become more significant. Sanchez et al. (2000), who evaluated skim milk and XG mixtures, also reported these findings [24].

During acid gel formation, the balance between aggregation/gelation of NaCAS and the segregative phase separation determines the acid gel microstructure and, as a result, its hardness. As C_XG_ increases, the elasticity of the gel network becomes higher (Figure 5B).

On the other hand, when the gelation process is slower, the gel network can be compacted as a result of the rupture of some interactions and formation of new ones, resulting in a more compact gel mesh. Cavallieri and da Cunha (2008) have also reported that the gelation rate can affect the hardness and elasticity of protein gels [30]. As R decreases, the rate at which pH becomes lower decreases, so the formation of NaCAS-XG acid gel network is retarded and the gel network elasticity increases (Figure 5B).

### 2.5. Microstructure of NaCAS Acid Gels in the Presence of XG

**Figure 6:**
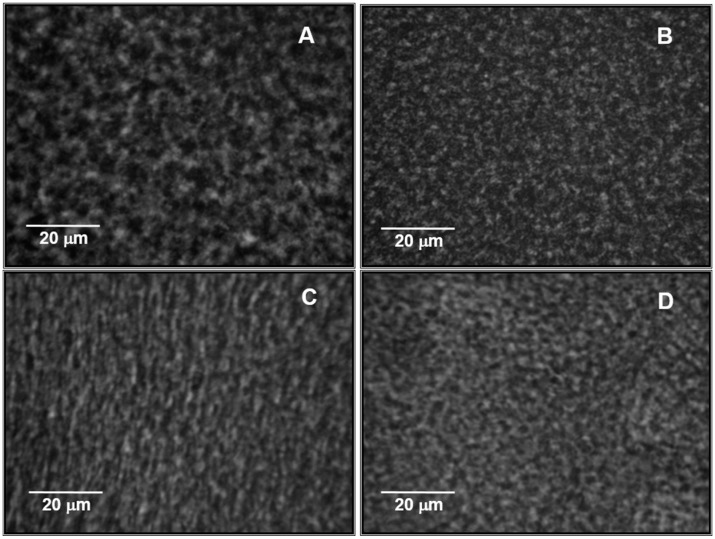
Digital images of acid gels, *C*_NaCAS_ 3 wt %, *R* 0.5 and *T* 25 °C: (**A**) without GX; (**B**) *C*_GX_ 0.10 wt %; (**C**) *C*_GX_ 0.15 wt % and (**D**) C_GX_ 0.20 wt %; oil immersion objective of 100× on an inverted microscope which was coupled to a digital camera with a 52 mm adaptor and 9.1× zoom.

Figure 6 shows the digital images of the microstructure of NaCAS-XG gels obtained. The dark zones represent the pores or interstices and the white zones represent the protein gel network.

Table 3 shows the average diameter obtained for the pores or interstices of NaCAS-XG acid gels. In the presence of XG, the average pore diameters decreased, i.e., the degree of compactness of NaCAS-XG gels increased. This effect increases with an increment of *C*_XG_, in agreement with *G*’_max_ values, since the smaller the pore size, the greater the elasticity of the gel network. In addition, as C_XG_ increases, the hydrophobic interactions, which are involved in the compaction process of the NaCAS gel network, increase because the hydrophobic residues are more exposed, as it was verified by spectrofluorimetric determinations. Rohart and Michon (2014) also reported changes on the microstructure of skim milk gels as *C*_XG_ increases [31].

## 3. Conclusions

Above the isoelectric point of NaCAS, the addition of XG leads to a segregative phase separation throughout the range of the concentrations assayed. During the acid gelation process, near the protein isoelectric point, attractive interactions between both biopolymers may occur.

In addition, the presence of the polysaccharide leads to NaCAS conformational changes related to a higher exposition of the protein intrinsic fluorophores to a polar environment as the C_XG_ increases. This fact favors the hydrophobic interactions between NaCAS particles during gel formation. The presence of this polysaccharide modifies the kinetics of the gelation process and induces the formation of more compact gels with a high elastic component.

The gel microstructure depends on the balance among thermodynamic incompatibility, NaCAS gelation and protein-XG interactions. According to the XG concentration added, gels with different textures can be obtained. These findings could contribute to the design of milk-protein based food.

## 4. Materials and Methods

### 4.1. Materials

Bovine sodium caseinate powder (NaCAS), glucono-δ-lactone (GDL), xanthan gum (XG), tris(hydroxymethyl)aminomethane (Tris) were purchased from Sigma-Aldrich Co. (Steinheim, Germany), and used without further purification. HCl and NaOH were provided by Cicarelli SRL (San Lorenzo, Argentina).

NaCAS and XG aqueous stock suspensions, 10 wt % and 1 wt % respectively, were prepared in distilled water at room temperature. For thermodynamic compatibility assays, protein and polysaccharide solutions were prepared in buffer Tris HCl 10 mM, pH 6.80. Protein concentration was determined by the Kuaye’s method [32].

### 4.2. Phase Diagrams

To evaluate the thermodynamic compatibility between both biopolymers, binary solutions of NaCAS-XG were prepared by carefully mixing weighed amounts of NaCAS (10 wt %) and XG (1 wt %) in buffer Tris HCl 10 mM, pH 6.80, at room temperature.

The phase diagrams or binodals were obtained using the method proposed by Spyropoulos et al. (2010) [33]. Polysaccharide/protein aqueous solutions were prepared to give rise to binary systems. On one hand, polysaccharide concentration remained constant while protein concentrations ranged from 0 to 4 wt %; on the other hand, protein concentration remained constant while polysaccharide concentrations ranged from 0 to 0.45 wt %. From these binary solutions, two samples were taken and kept in a humidity chamber (at 25 or 35 °C and 40% humidity), for 24 or 48 h (*n* = 2). The occurrence of phase separation or the lack of it was verified by visual inspection.

### 4.3. Intrinsic Fluorescence Emission Spectra

In order to detect changes in the relative intensity of fluorescence (FI) and/or any spectral shift, associated with possible conformational changes of the protein studied, excitation and emission spectra of NaCAS (0.1 wt %) in the absence or presence of different concentration of XG were obtained. Previously, the excitation wavelength (λ_exc_) and the range of protein concentration with a negligible internal filter effect were determined. Samples (3 mL) for spectral analysis were poured into a fluorescence cuvette (1 cm path length) and placed into a cuvette holder maintained at 35 °C. Values of FI (*n* = 3) were registered within the range of 300 to 400 nm using a λ_exc_ of 291 nm.

### 4.4. Viscometry

The gelation process is limited by particle diffusion, which depends on medium viscosity (η). Therefore, it is essential to evaluate the effect that the addition of XG, in the absence of protein, exerts on that property. η was measured, using a rotational LV Master (LVDV-III) viscosimeter Brookfield (Brookfield Engineering Laboratories, Middleboro, MA, USA) with cone/plate geometry (CPE-42), thermostatically controlled at 35 °C and a shear rate of 11.54 s^−1^ (*n* = 3). The relative viscosity (η_r_) was calculated as:

(4) $$\eta_{r}\;=\;\frac{\eta }{\eta_{0}}$$

where η is the solution viscosity and η_0_ is the solvent viscosity.

### 4.5. Rheological Properties of Acid Gels—Experimental Design

Rheological properties of NaCAS samples (3 and 5 wt %), in the absence or presence of XG (0.10 and 0.20 wt %), were determined. A stress and strain controlled AR G2 model rheometer (TA Instruments, New Castle, DE, USA) using a cone geometry (diameter: 40 mm, cone angle: 2°, cone truncation: 55 mm) and a system of temperature control (15 and 35 °C) with a recirculating bath (Julabo model ACW 100, Seelbach, Germany) connected to a Peltier plate was used. The amount of GDL to be added was calculated using the following relation (R):

(5) $$R\;=\;\frac{\mathrm{GDL}\;\mathrm{mass}\;\mathrm{fraction}\;}{\mathrm{NaCAS}\;\mathrm{mass}\;\mathrm{fraction}\;}$$

In order to start the acid gelation process, an amount of solid GDL according to a certain *R* (0.35 or 1) was added. Measurements were performed every 20.8 s for 120–180 min with a constant oscillation stress of 0.1 Pa and a frequency of 0.1 Hz. To ensure that the measurements of storage or elastic modulus (G’) and the loss or viscous modulus (G’’) were always obtained within the linear viscoelastic region, the Lissajous figures at various times were plotted.

The G’–G’’ crossover times of acidified systems were considered here as the gel times (t_gel_) according to previous studies of milk/caseinate gelation [29,34]. The pH at t_gel_ (pH_gel_) was determined considering the pH value at the G’–G’’ crossover. In addition, the maximum storage modulus (G’_max_) was determined (*n* = 2).

To evaluate the significance of the effects of independent variables *T* (15 and 35 °C), *R* (0.35 and 1), *C*_XG_ (0, 0.10 and 0.20 wt %) and concentration of NaCAS (C_NaCAS_) (3 and 5 wt %) on the dependent variables t_gel_, pH_gel_ and maximum elasticity of gel mesh (G’_max_), a fractional factorial design 2^4-1^ was carried out. The significant factors and interactions were evaluated by ANOVA. Using the corresponding models, the responses were adjusted and surface plots were performed for each situation.

### 4.6. Conventional Optical Microscopy (OCM)

The degree of compactness of the gel network was evaluated through digital image analysis. Stock solutions of NaCAS were prepared and subsequently mixtures of NaCAS (3 wt %) and XG (0 to 0.20 wt %) were performed. The gelation was started by adding solid GDL (*R* = 0.5) to 3 g of samples (*n* = 4). After GDL addition, each sample (90 μL) was immediately placed in compartments of LAB-TEK II cells (Thermo Scientific, Waltham, MA, USA). The gelation reaction was performed in an oven at 25 °C, keeping the humidity controlled. Gels were observed with an oil immersion objective of 100× on an inverted microscope (Union Optical Co. Ltd., Tokyo, Japan) which was coupled to a digital camera (Canon Powershot A640, Buenos Aires, Argentina) with a 52 mm adaptor and 9.1× zoom. Acquired images were stored in JPG format for their further analysis. The mean diameter of pores or interstices was determined through Image J software (Biotechnology and Biological Science Research Council, Swindon, UK), which was obtained in pixel units. By means of a micrometer rule, it was determined that 1 pixel = (0.0399 ± 0.0001) μm. Then, the image resolution in this optical system was found to be 25.0 pixels/μm.

### 4.7. Statistical Analysis

Data presented are average values ± standard deviations. Statistical analysis was performed with Sigma Plot 10.0 (Systat Software Inc., San José, CA, USA) and Minitab 16 software (Minitab Statistical Software, State College, PA, USA). The relationship among variables was evaluated by correlation analysis, using the Pearson correlation coefficient (p). Differences were considered statistically significant at *p* < 0.05 values. Small *p*-values imply that the effects (or coefficients) are much greater than their standard error [35].

## Figures and Tables

**Table 1:** Gelation times (t_gel_), gelation pH (pH_gel_) and maximum elastic modulus (G’_max_) as a function of the coded values for sodium caseinate concentrations (C_NaCAS_), xanthan gum concentrations (C_XG_), temperature (T) and GDL mass fraction/NaCAS mass fraction ratio (R) used in the experimental design, with the respective real values.

Independent variables				Responses		
C_NaCAS_ (wt %)	C_XG_ (wt %)	R	T (°C)	t_gel_ (min) ^a^	pH_gel_^a^	G’_max_ (Pa) ^b^
3 (−1)	0.2 (+1)	1.00 (+1)	15 (−1)	3.04	6.11	1070.00
3 (−1)	0.1 (0)	0.35 (−1)	15 (−1)	26.92	5.77	1346.00
3 (−1)	0 (−1)	1.00 (+1)	15 (−1)	17.53	5.17	109.30
3 (−1)	0 (−1)	0.35 (−1)	15 (−1)	76.04	4.97	228.20
5 (+1)	0.2 (+1)	0.35 (−1)	15 (−1)	3.39	6.39	2733.00
5 (+1)	0.1 (0)	1.00 (+1)	15 (−1)	10.90	5.59	1236.00
5 (+1)	0 (−1)	0.35 (−1)	15 (−1)	68.00	5.12	781.80
5 (+1)	0 (−1)	1.00 (+1)	15 (−1)	19.99	4.95	495.00
3 (−1)	0.2 (+1)	0.35 (−1)	35 (+1)	3.23	6.18	2496.25
3 (−1)	0.1 (0)	1.00 (+1)	35 (+1)	5.33	5.65	218.65
3 (−1)	0 (−1)	1.00 (+1)	35 (+1)	6.95	4.95	26.87
3 (−1)	0 (−1)	0.35 (−1)	35 (+1)	19.59	5.39	22.71
5 (+1)	0.2 (+1)	1.00 (+1)	35 (+1)	3.62	5.92	1400.00
5 (+1)	0.1 (0)	0.35 (−1)	35 (+1)	14.61	5.49	484.30
5 (+1)	0 (−1)	1.00 (+1)	35 (+1)	6.65	4.94	90.83
5 (+1)	0 (−1)	0.35 (−1)	35 (+1)	17.89	5.27	116.30

^a^ Mean value ± 0.02; ^b^ Mean value ± 0.01.

**Table 2:** Analysis of the coefficients and *p*-values obtained for the responses t_gel_. pH_gel_ and G’_max_.

Factor	t_gel_ (min)		pH_gel_		G’_max_ (Pa)	
	Coefficient	*p*-Value	Coefficient	*p*-Value	Coefficient	*p*-Value
Constant	15.60	0.000	5.63	0.000	1002.2	0.000
C_NaCAS_ (L)	-	― ^a^	-	― ^a^	-	― ^a^
C_XG_ (L)	−12.86	0.000	0.51	0.000	832.1	0.000
R (L)	−7.54	0.002	−0.09	0.024	−290.3	0.011
T (L)	−7.23	0.002	-	― ^a^	-	― ^a^
C_NaCAS_ × C_NaCAS_ (Q)	-	― ^a^	-	― ^a^	-	― ^a^
C_XG_ × C_XG_ (Q)	-	― ^a^	-	― ^a^	-	― ^a^
R × R (Q)	-	― ^a^	-	― ^a^	-	― ^a^
T × T (Q)	-	― ^a^	-	― ^a^	-	― ^a^
C_NaCAS_ × C_XG_	-	― ^a^	-	― ^a^	-	― ^a^
C_NaCAS_ × R	-	― ^a^	-	― ^a^	-	― ^a^
C_NaCAS_ × T	-	― ^a^	-	― ^a^	-	― ^a^
C_XG_ × T	8.72	0.001	-	― ^a^	-	― ^a^
C_XG_ × R	8.16	0.002	-	― ^a^	−301.3	0.019
R × T	5.78	0.006	-	― ^a^	-	― ^a^
		*r*^2^ = 89.90%		*r*^2^ = 90.79%		*r*^2^ = 79.91%

L = linear effect; Q = quadratic effect; ― ^a^ Not significant.

**Table 3:** Average diameter of the pores of gels of *C*_NaCAS_ 3 wt %; *C*_XG_ variable; *R* 0.5 and *T* 25 °C.

*C*_XG_ (wt %)	Average Diameter of Pores (μm)
0	4.57 ± 0.04 ^a^
0.10	4.30 ± 0.09
0.15	4.07 ± 0.04
0.20	3.98 ± 0.04

^a^ Mean value ± standard deviation (*p* < 0.05).
